# Three *Brachypodium distachyon* Uev1s Promote Ubc13-Mediated Lys63-Linked Polyubiquitination and Confer Different Functions

**DOI:** 10.3389/fpls.2016.01551

**Published:** 2016-10-18

**Authors:** Huiping Guo, Rui Wen, Qianqian Wang, Raju Datla, Wei Xiao

**Affiliations:** ^1^College of Life Sciences, Capital Normal UniversityBeijing, China; ^2^National Research Council Canada, SaskatoonSK, Canada; ^3^Department of Microbiology and Immunology, University of Saskatchewan, SaskatoonSK, Canada

**Keywords:** *Brachypodium distachyon*, Uev1, Ubc13, K63-linked polyubiquitination, DNA-damage response

## Abstract

In this study, we report the identification and functional characterization of three *Brachypodium distachyon UEV* genes. All three BdUev1s form heterodimers with BdUbc13s, which are capable of catalyzing Lys63-linked polyubiquitination *in vitro*. The three *BdUEV1* genes are also able to functionally complement the budding yeast *mms2* mutant defective in DNA-damage tolerance. BdUev1A differs from the other two BdUev1s in that it contains an 18-amino acid tail, which appears to compromise its function in yeast, as deletion of this tail restores full function. BdUev1A is excluded from the nucleus, whereas BdUev1B, BdUev1C and the C-terminally truncated BdUev1A are mainly found in the nucleus. These and the *BdUEV1* gene expression analysis allow us to speculate that although all three BdUev1s function by promoting Lys63-linked polyubiquitination, BdUev1B and BdUev1C are involved in DNA-damage response and possibly other nuclear functions, while BdUev1A is required for non-nuclear function(s).

## Introduction

Ubiquitin (Ub) is a highly conserved 76-residue protein; its amino acid sequence is conserved in animals, plants, and fungi (Hochstrasser, 1996; Hershko and Ciechanover, 1998). One ubiquitin C terminus can be covalently linked to any one of the seven surface lysine residues (K6, K11, K27, K29, K33, K48, and K63) or the N-terminal methionine residue of a second ubiquitin via isopeptide bond, forming a ubiquitin chain. Substrates can be modified by ubiquitin chains (polyubiquitination), multiple single ubiquitin (multi-monoubiquitination) or a single ubiquitin (monoubiquitination). Ub-activating enzyme (Uba or E1), Ub-conjugating enzyme (Ubc or E2) and Ub ligase (E3) are involved in this process. Lys48-linked chains are the predominant linkage type, and play a role in targeting protein for degradation by the 26S proteasome (Hershko and Ciechanover, 1998). Lys63-linked chains are the second abundant form and their role is to target proteins to primarily serve as a signal (Pickart, 2000; Chen and Sun, 2009). Ubc13 is the only known Ubc dedicated to catalyzing Lys63-linked polyubiquitination reaction in eukaryotes. This reaction requires a Ubc-like or Ubc/E2 variant (Uev), which forms a stable heterodimer with Ubc13 (Hofmann and Pickart, 1999; McKenna et al., 2001).

The first *UEV* gene, denoted *MMS2*, was isolated from budding yeast cells and is involved in the error-free DNA-damage tolerance (DDT) pathway (Broomfield et al., 1998; Xiao et al., 1999). Mms2 itself is homologous to an E2 enzyme, but lacks the active site cysteine residue (Broomfield et al., 1998). Several structural studies revealed that Mms2 is required for catalyzing Lys63-linked poly-Ub chains (Moraes et al., 2001; VanDemark et al., 2001). In budding yeast, Mms2, Ubc13 and Rad5 form a E2-E3 complex and are all required for the sequential Lys63-linked polyubiquitination of PCNA after it is monoubiquitinated at the Lys164 residue by another E2–E3 complex, Rad6-Rad18 (Hoege et al., 2002). Yeast has only one *UEV* (*MMS2*) gene (Broomfield et al., 1998) while higher eukaryotic genomes appear to contain multiple *UEV* genes (Xiao et al., 1998; Wen et al., 2008, 2012) whose products form stable complexes with Ubc13 and may participate in different biological processes. For example, mammalian Uev1A-Ubc13 is involved in NF-κB activation, while Mms2-Ubc13 is required for DNA-damage response (Andersen et al., 2005). It has been reported that *Arabidopsis UBC13* genes are involved in DNA-damage response (Wen et al., 2006), apical dominance (Yin et al., 2007), iron metabolism (Li and Schmidt, 2010), immunity (Mural et al., 2013) and auxin signaling (Wen et al., 2014). In contrast, little is known about plant *UEV* genes. Among *Arabidopsis UEV1* genes, only *AtUEV1D* has been shown to be involved in DNA-damage response (Wen et al., 2008). So far there has been no report on the characterization of other plant *UEV1* genes.

*Brachypodium distachyon* is a model species for monocots, temperate cereals and biofuel plants (Draper et al., 2001; Catalan et al., 2014). It has many advantages including: small genome (300 Mbp), short generation time (8–12 weeks), relative convenience of getting mutants and simpler growth condition. We previously reported that the *B. distachyon* genome contains two *UBC13* genes; their products were able to promote Lys63-linked Ub chain assembly with AtUev1s and functionally rescue yeast *ubc13* mutant phenotypes (Guo et al., 2016). Here we report molecular cloning and functional characterization of three *UEV1* genes from *B. distachyon*. All three products can interact and catalyze Lys63-linked Ub chain assembly with BdUbc13; however, BdUev1B and BdUev1C appear to be involved in DDT while BdUev1A is not.

## Materials and Methods

### Plant Materials and Yeast Cell Culture

*B. distachyon 21* (Bd21) seeds were surface sterilized with 20% sodium hypochlorite twice for 10 min, rinsed five times with sterile H_2_O, incubated in H_2_O for 12 h at room temperature, and then transferred to a wet filter paper to germinate in darkness for 24 h at 22–25°C. Uniformly germinated seeds were spread on plastic pots containing 1/2 MS (Murashige and Skoog), and the medium was changed every 2 days. After 2 weeks, the seedlings were transferred to soil and grown in a growth chamber with a daily photo cycle of 16/8 h light/dark, 22°C/18°C and 65–75% air humidity.

Yeast strains used in this study are listed in Supplementary Table S1 and the cell culture conditions were as previously described (Guo et al., 2016). Yeast cells were transformed by a lithium acetate method (Ito et al., 1983). The *mms2Δ::HIS3* (Xiao et al., 1999) and *ubc13*Δ::HUH (Wen et al., 2008) disruption cassettes were as previously described.

### Molecular Cloning and Plasmid Construction

To clone *B. distachyon UEV1* genes, total RNA was extracted from *B. distachyon* seedlings using a Trizol reagent (Invitrogen), the first-strand of cDNA was synthesized by a RevertAid First Strand cDNA Synthesis Kit (Thermo). The *BdUEV1* ORFs were amplified by PCR from the above cDNAs using gene-specific primers (Supplementary Table S2). The yeast two-hybrid vectors pGAD424Bg and pGBT9Bg were derived from pGAD424 and pGBT9 (Bartel and Fields, 1995).

### Yeast Two-Hybrid and GST Pull-Down Assays

Constructed Gal4_AD_ and Gal4_BD_ vectors were transformed into the yeast two-hybrid strain PJ69-4a (James et al., 1996) in pairs and allowed to grow at 30°C for 2–3 days. Transformants were selected on SD-Leu-Trp plates. Protein interaction was determined on synthetic complete medium lacking Trp, Leu and His, supplemented with 3-amino-1,2,4-triazole (3-AT, Sigma-Aldrich), or on plates lacking Trp, Leu, and Ade. SD-Leu-Trp plates were used as a control.

Full-length *BdUEV1* ORFs were cloned in plasmid pGEX-6p and *BdUBC13*s were cloned in plasmid pET30a as previously described (Guo et al., 2016). The His_6_ and GST fusion constructs were transformed into *E. coli* strain BL21 (DE3) and the recombinant proteins were purified with Ni Sepharose and glutathione (Amersham Pharmacia), respectively. For the pull-down assay, crude cell extracts were loaded on Glutathione Sepharose^TM^ 4B beads and then 10 μg of purified His_6_-BdUbc13 was later added. After incubation and washing, the GST beads were boiled with SDS-PAGE loading buffer for 10 min prior to Western blotting.

### Ub Conjugation Reaction

Ub conjugation reactions were performed by using purified fusion proteins and Ub thioester conjugation reagents (Boston Biochem). The reaction mixture contained 225 nM E1 enzyme, 200 μM Ub (or recombinant Ub-K63R), 1 mM MgATP, 1 mM Ubc13 and 1 mM Uev1 in 20 μl of reaction buffer. The conjugation reactions were performed at 37°C for 2 h, samples were subjected to 12% SDS-PAGE and Ub-containing molecules were detected by Western blotting using the mouse anti-Ub monoclonal antibody P4D1 (Cell Signaling).

### Yeast Survival Assays

Yeast strain HK578-10D, its isogenic *mms2Δ* single and *ubc13Δ mms2Δ* double mutants were transformed with pGAD-BdUEV1s, or co-transformed with pGBT-BdUEV1 and pGAD-BdUBC13. Transformants were selected on SD-Leu or SD-Leu-Trp plates. The gradient plate and serial dilution assays were as described (Xu et al., 2014).

### Spontaneous Mutagenesis Assay

Spontaneous mutagenesis was measured by the reverse mutation rate of the *trp1-289* allele in the DBY747 strain (Xiao and Samson, 1993). DBY747 cells with the *mms2Δ* mutation were transformed with pGAD-BdUEV1s and the transformants were selected on SD-Leu plates. This modified Luria–Delbruck fluctuation test was adapted as described (Blackwell et al., 2014) and each treatment contained five independent cultures.

### Subcellular Localization of BdUev1s

*GFP-BdUEV1* fusion genes were constructed by cloning *BdUEV1* ORFs into the modified binary vector pCAMBIA1300-GFP downstream of *GFP* at the *Sac*I and *Kpn*I sites so that the fusion gene was driven by a CaMV 35S promoter. The constructed plasmids were introduced into *A. tumefaciens* PMP90, which was then co-infiltrated with *A. tumefaciens* P19 into *N. benthamiana* leaves (Waadt and Kudla, 2008). GFP and DAPI fluorescence was observed 2 days after transformation using a Zeiss 780 confocal microscope.

### Expression Analysis by Droplet Digital RT-PCR (ddRT-PCR)

To determine the expression level of *BdUEV1* genes in different development stages and in response to abiotic stress, ddRT-PCR was performed by using a Bio-Rad QX200^TM^ ddPCR System. All reagents and consumables for the experiments including droplet generator oil, DG8^TM^ cartridges and gaskets, droplet reader oil, and ddPCR supermix for EvaGreen were purchased from Bio-Rad. Briefly, the Droplet Digital PCR began by partitioning the reaction mix containing EvaGreen Supermix, primers and sample cDNA into aqueous droplets in oil via the QX200 Droplet Generator; after transfer of droplets to a 96-well PCR plate, a thermocycling protocol including 95°C for 5 min; 95°C for 30 s, 40 cycles; 60°C for 60 s, 40 cycles (ramp rate set to 2°C/s); 4°C for 5 min; 90°C for 5 min and 4°C infinite was carried out in a conventional thermal cycler. The PCR plate was then transferred to the QX200 Droplet Reader for automatic reading of samples. QuantaSoft^TM^ Software was used for data analysis. The primers used to amplify the *BdUEV1*s are listed in Supplementary Table S3.

## Results

### Identification and Sequence Analysis of the *BdUEV1* Genes

**FIGURE 1:**
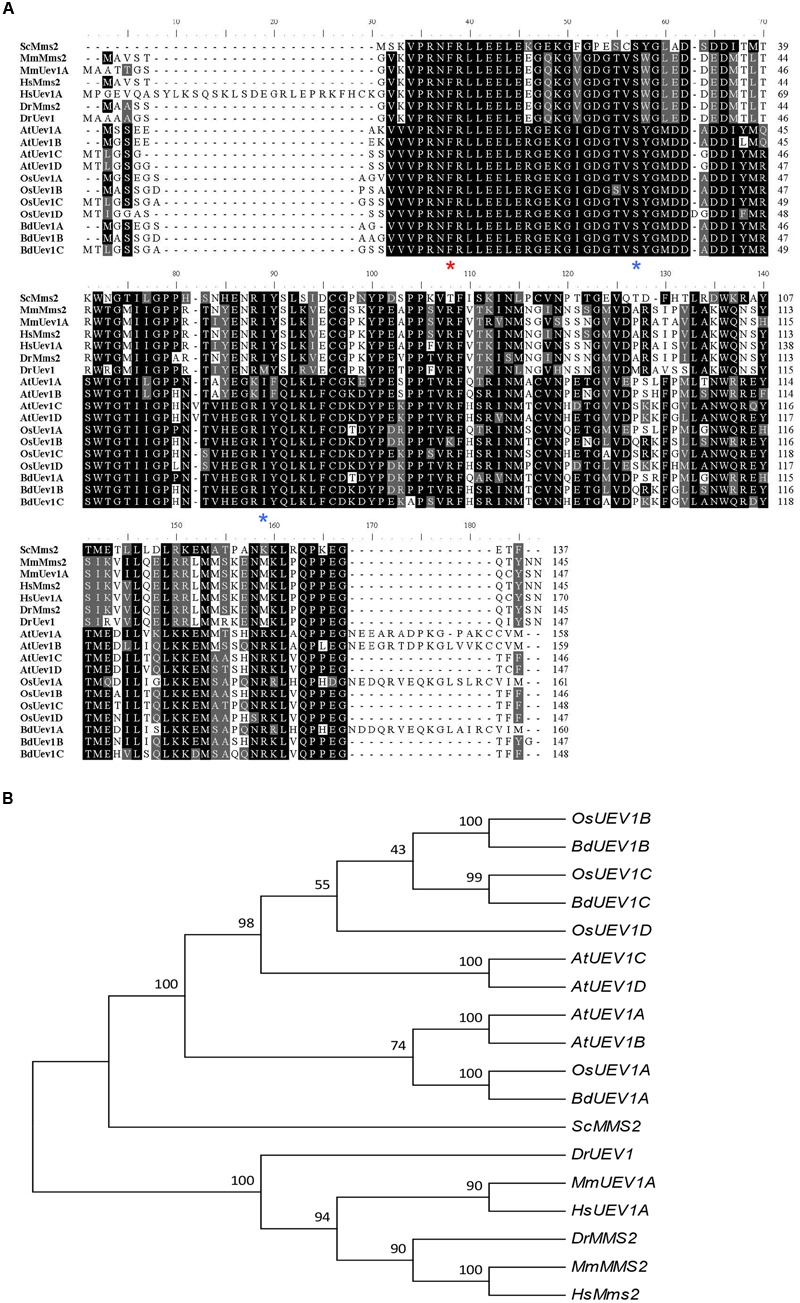
**Analysis of *UEV1s* from different organisms. (A)** Amino acid sequence alignment of BdUev1s and Uevs from other organisms. The sequences were aligned and edited using BioEdit 7.0.9. Critical residues for UEV functions are indicated with asterisks underneath the residue. Sc, *S. cerevisiae* (NP_011428); Mm, *Mus musculus* (*MmMMS2* = NP_076074, *MmUEV1A* = NP_075719.1); Hs, *Homo sapiens* (*HsMMS2* = NP_003341.1, *HsUEV1A* = NP_068823.2); Dr, *Danio rerio* (*DrMMS2* = NP_998680.1, *DrUEV1* = NP_001032479); At, *A. thaliana* (*AtUEV1A* = NP_564191, *AtUEV1B* = NP_564994, *AtUEV1C* = NP_850259, *AtUEV1D* = NP_001190073); Os, *Oryza sativa* Japonica Group (*OsUEV1A* = XP_015632881, *OsUEV1B* = XP_015620277, *OsUEV1C* = XP_015611980, *OsUEV1D* = XP_015635908); Bd, *B. distachyon* (*BdUEV1A* = KQK13622, *BdUEV1B* = XP_003577901, *BdUEV1C* = XP_003577953). **(B)** Phylogenetic analyses of *UEV* family CDSs from different organisms. The phylogenetic tree clustering was conducted by using MEGA6.0.

To identify *B. distachyon UEV1* genes, *Arabidopsis UEV1* genes were used to blast the *B. distachyon* genomic database. Three genes, *BRADI1G11410.1, BRADI4G02400.1* and *BRADI4G27530.1*, were obtained and named *BdUEV1A, BdUEV1B* and *BdUEV1C*, respectively. Protein sequence alignment of BdUev1s reveals 81% identity between BdUev1A and BdUev1B, 77% identity between BdUev1A and BdUev1C, and 82% identity between BdUev1B and BdUev1C. The predicted BdUev1A, BdUev1B and BdUev1C proteins contain 160, 147 and 148 amino acids, respectively. It is noted that BdUev1A contains a C-terminal tail but the other two BdUev1s do not (**Figure 1A**). The sequences of BdUev1 proteins were also aligned with those of Uev1 proteins from six other eukaryotic organisms. As shown in **Figure 1A**, several critical residues deemed to be necessary for the Uev activity are also conserved in BdUev1s, including Phe13 of hMms2 (red asterisk) required for the physical interaction with Ubc13 (Pastushok et al., 2005), and Ser27 and Ile57 of ScMms2 (blue asterisks) required for the non-covalent interaction with Ub and poly-Ub chain assembly (Tsui et al., 2005; Eddins et al., 2006; Pastushok et al., 2007).

To further investigate the evolution of *B. distachyon UEV1*s, the CDS sequences of three *BdUEV1*s were compared with *UEV*s from six other organisms. The analysis reveals that *BdUEV1*s and *OsUEV1*s are closely related (**Figure 1B**). Hence, *BdUEV1*s could be another ideal model to study plant *UEV1* gene functions, especially to study crop *UEV1* gene functions.

### Interaction of BdUev1s with BdUbc13s

**FIGURE 2:**
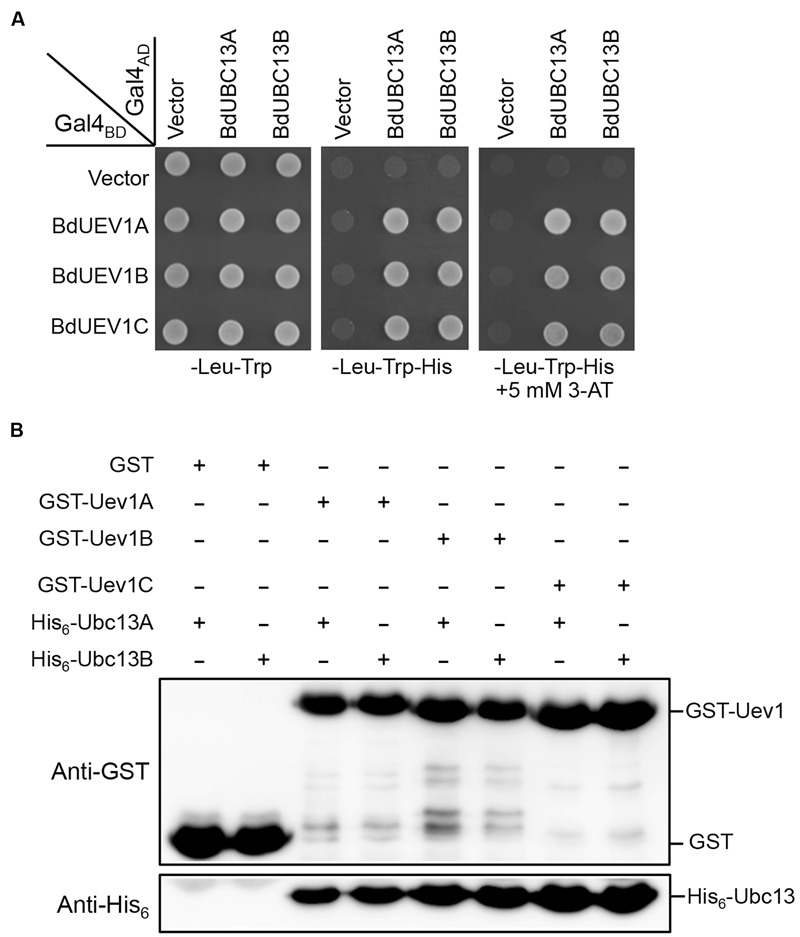
**BdUev1s physically interact with BdUbc13s. (A)** Physical interaction between BdUev1s and BdUbc13s in a yeast two-hybrid assay. PJ69-4a cells were transformed with *BdUEV1*s and *BdUBC13*s genes and the transformants carrying one Gal4_BD_ (pGBT9) and one Gal4_AD_ (pGAD424) plasmid were then selected, replicated onto various plates as indicated and incubated for 3 days before being photographed. The result is representative of at least five independent transformants from each treatment. **(B)** Protein interactions between BdUev1s and BdUbc13s by an affinity pull-down assay. BL21 (DE3) cells were transformed with pGEX-BdUEV1s, and target gene expression was induced by adding 0.2 mM IPTG. Crude cell extracts were loaded on Glutathione Sepharose^TM^ 4B beads and 10 μg of purified His_6_-BdUbc13.

Since Uevs play an essential role in Ubc13-mediated Lys63-linked polyubiquitination through forming a stable complex with Ubc13 to maintain its unique E2 activity (Hofmann and Pickart, 1999; McKenna et al., 2001, 2003), a yeast two-hybrid assay (Fields and Song, 1989) was used to determine whether BdUev1s interact with BdUbc13s. As seen in **Figure 2A**, all three BdUev1s interact with both BdUbc13s and the co-transformants are able to grow in the presence of 5 mM 3-AT, while no self-activation is observed in the -His plate without 3-AT. To further confirm the physical interaction between BdUev1s and BdUbc13s *in vitro*, a GST-affinity pull-down assay was performed. As shown in **Figure 2B**, recombinant GST-BdUev1 fusion proteins can pull down purified recombinant His_6_-BdUbc13 proteins, but the GST alone cannot. In the GST-affinity pull-down assay, the strength of interaction with BdUbc13 has no detectable difference. Based on the above observations, we conclude that BdUev1s can form stable heterodimers with BdUbc13s *in vitro*.

### BdUev1 Is Required for Ubc13-Mediated Lys63-Linked Polyubiquitination *In vitro*

**FIGURE 3:**
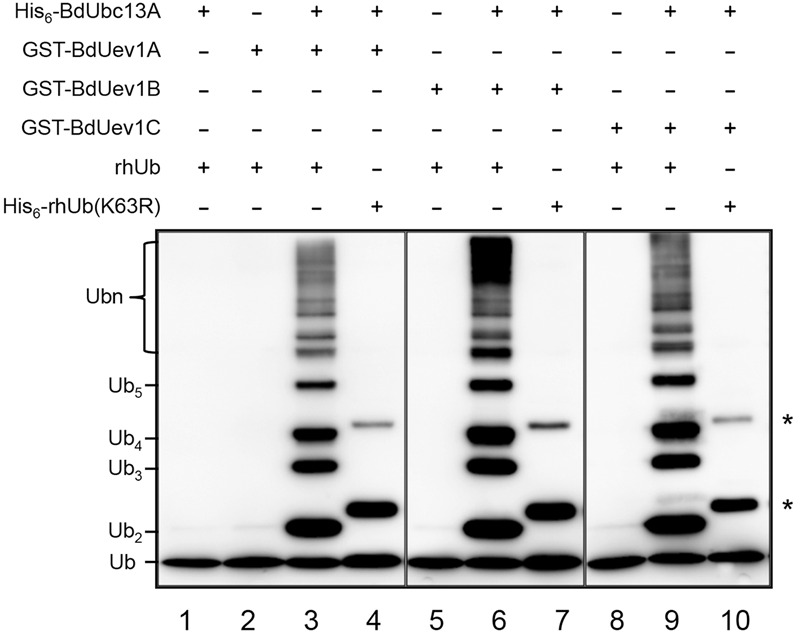
***In vitro* ubiquitin conjugation assay of BdUev1s using purified proteins.** After ubiquitination reactions as described, samples were subjected to SDS-PAGE and Western blotting using an anti-Ub antibody to monitor poly-Ub chain formation. rhUb, recombinant human ubiquitin. Asterisks indicate the non-specific bands in lanes 4, 7, and 10.

So far, the only defined Uev activity is to promote Lys63-linked polyubiquitination along with Ubc13 (Hofmann and Pickart, 1999; Deng et al., 2000; Hoege et al., 2002). A standard means of characterizing Uev enzymatic activity is by an *in vitro* Ub conjugation assay (Hofmann and Pickart, 1999). To avoid Ub conjugation to Ubc13 itself (McKenna et al., 2001), we made the corresponding BdUbc13-K94R mutation that does not affect the free Ub chain formation (Guo et al., 2016). As shown in **Figure 3**, BdUev1s together with BdUbc13A can indeed generate free Ub chains (lanes 3, 6, and 9), while BdUbc13A (lane 1) or BdUev1s (lanes 2, 5, and 8) alone cannot. More importantly, when the Ub-K63 is mutated to R, there are no poly-Ub chains observed in the same reaction (lanes 4, 7, and 10, asterisks indicate the background of UbK63R, see Supplementary Figure S1, lane 3). Hence, we conclude that BdUev1s are indeed required for Lys63-linked poly-Ub chain assembly. Since it has been previously reported that both BdUbc13A and BdUbc13B have the same ability to promote Lys63-linked poly-Ub chains along with AtUevs (Guo et al., 2016), this study did not repeat the reaction with BdUbc13B.

### Functional Complementation of Yeast *mms2* by *BdUEV1s*

**FIGURE 4:**
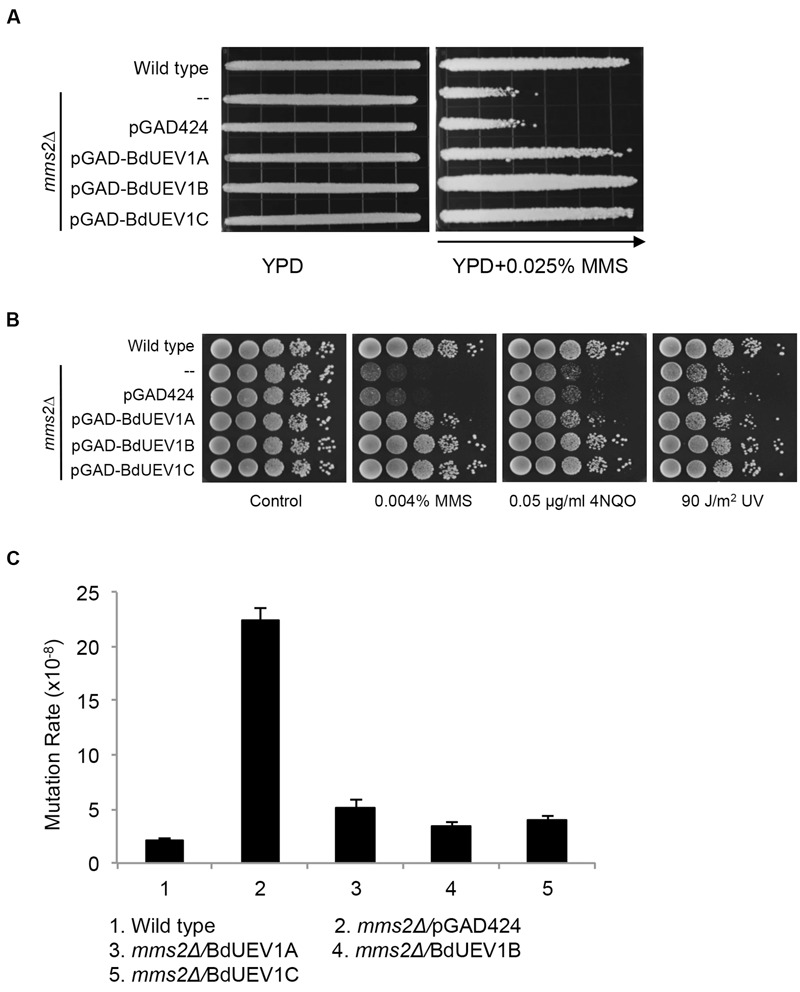
**Functional complementation of the yeast *mms2* null mutant by *BdUEV1*s. (A)** Complementation of the *mms2* single mutant by *BdUEV1*s. Yeast strain HK578-10D (wild type) and the isogenic *mms2Δ* transformants were grown overnight and then printed onto the gradient plate. The YPD control (left) and YPD+0.025% MMS gradient (right) plates were incubated at 30°C for 3 days. Arrow points to increasing MMS concentrations. **(B)** Functional complementation of the *mms2* null mutant by BdUev1A, BdUev1B and BdUev1C using representative DNA-damaging agents by a serial dilution assay. Yeast strains as indicated were grown overnight in SD selective media, diluted and treated with the DNA-damaging agents. Yeast strains used: HK578-10D (wild type) and WXY942 (*mms2*Δ). **(C)** Spontaneous mutation rates of *S. cerevisiae mms2* mutants. All strains are isogenic derivatives of DBY747. The results are the average of three independent experiments with standard deviations. All the genes were cloned in pGAD424.

Yeast Mms2 is a member of the error-free DDT pathway (Broomfield et al., 1998; Brusky et al., 2000). To determine whether BdUev1s also play critical roles in error-free DDT, we assessed the ability of *BdUEV1*s to rescue the yeast *mms2* mutant phenotypes. As shown in **Figure 4A**, the expression of any one of the three *BdUEV1* genes can protect the *mms2* null mutant from killing by MMS, whereas the expression of pGAD424 vector cannot. It is interesting that the ability of *BdUEV1A* to rescue the *mms2* null mutant is slightly less than other two *BdUEV1*s.

DNA damage induced by MMS, UV irradiation or 4-nitroquinoline 1-oxide (4NQO) is largely considered to cause replication blocks and the *mms2* null mutant is hypersensitive to these agents (Broomfield et al., 1998; Xu et al., 2013). To further confirm that *BdUEV1*s function in the DDT pathway, we performed a serial dilution assay in the presence of several representative DNA-damaging agents as mentioned above. A serial dilution assay is a semi-quantitative method to assess the relative sensitivity of cells to treatment (Xu et al., 2014). As shown in **Figure 4B**, both *BdUEV1B* and *BdUEV1C* can fully protect the yeast *mms2* null mutant from killing by MMS, UV or 4-NQO, while the protection provided by *BdUEV1A* appears to be compromised.

Loss of *MMS2* dramatically increases spontaneous mutagenesis, which serves as a hallmark indicating that it is involved in the error-free DDT pathway (Xu et al., 2015). Under our experimental conditions, deletion of *MMS2* caused a 10-fold increase in the spontaneous mutation rate. When the mutant cells were transformed with *BdUEV1*s, the spontaneous mutagenesis rate was reduced to less than twofold (**Figure 4C**). Again *BdUEV1B* and *BdUEV1C* appear to be more effective than *BdUEV1A* in complementing the yeast *MMS2* function. Hence, our results indicate that BdUev1B and BdUev1C can fully replace the function of Mms2 in the yeast error-free DDT pathway, but BdUev1A is partially functional.

**FIGURE 5:**
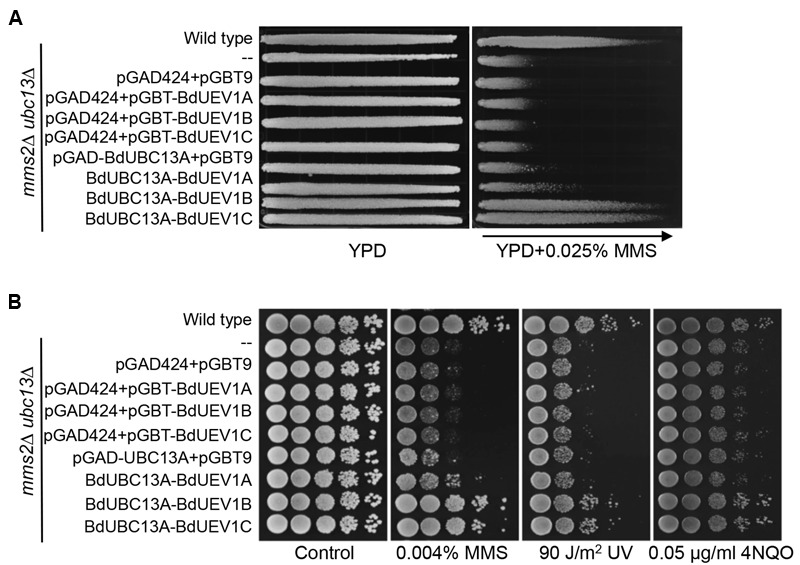
**Functional complementation of the yeast *ubc13 mms2* null mutant by *BdUEV1*s and *BdUBC13A*. (A)** Complementation of the *ubc13 mms2* double mutant by *BdUEV1*s and *BdUBC13A*. Yeast strain HK578-10D (wild type) and the isogenic *ubc13Δ mms2Δ* transformants were grown overnight and then printed onto the gradient plate. The YPD control (left) and YPD+0.025% MMS gradient (right) plates were incubated at 30°C for 3 days. Arrow points to increasing MMS concentration. **(B)** Functional complementation of the *ubc13 mms2* double mutant by *BdUBC13A* and *BdUEV1*s using representative DNA-damaging agents by a serial dilution assay. Yeast strains as indicated were grown overnight in SD selective media, diluted and treated with the DNA-damaging agents.

The above functional complementation studies require that BdUev1s form stable heterodimers with yeast Ubc13. To further confirm that BdUev1 and BdUbc13 can form a stable heterodimer to replace the function of both yeast Mms2 and Ubc13, we co-transformed yeast *ubc13 mms2* double mutant cells with the combination of *BdUEV1*s and *BdUBC13*s and assessed their relative tolerance to DNA-damaging agents. As shown in **Figure 5** and Supplementary Figure S2, expression of *BdUEV1* or *BdUBC13* alone cannot rescue the *mms2 ubc13* double mutant, whereas co-expression of *BdUEV1B* or *BdUEV1C* with *BdUBC13* can rescue the *ubc13 mms2* double mutant to nearly the wild-type level, regardless of the source of DNA damage. In sharp contrast, *BdUBC13A* or *BdUBC13B* co-transformed with *BdUEV1A* did not effectively rescue the *ubc13 mms2* double mutant from killing induced by MMS, UV or 4NQO.

### The C-terminal Tail of BdUev1A Determines Its Function in Yeast

**FIGURE 6:**
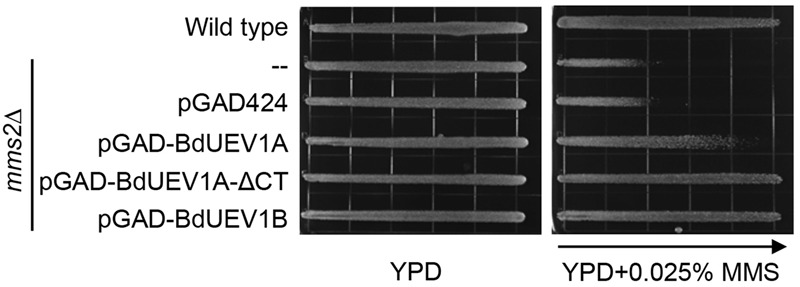
**The C-terminal tail of BdUev1A affects functional complementation of the yeast *mms2* null mutant.** *mms2* null mutant (WXY942) transformants with plasmids expressing BdUev1A, BdUev1A-ΔCT or BdUev1B were assessed by an MMS gradient plate assay. The plates were incubated at 30°C for 3 days. Arrow points to gradually increasing MMS concentration.

Among the three BdUev1s, an obvious sequence difference is the additional 18-amino acid tail found at the C terminus of BdUev1A (**Figure 1A**). We hypothesized that the lack of complementation of the yeast *mms2* mutant by *BdUEV1A* is due to its encoded C-terminal tail. To test this hypothesis, we created a *BdUEV1A-ΔCT* construct in which the C-terminal 18 amino-acid coding sequence is removed from *BdUEV1A*. **Figure 6** shows that *BdUEV1A-ΔCT* provides stronger protection against DNA damage than *BdUEV1A* and is comparable with that of *BdUEV1B*. Hence, the C-terminal tail of BdUev1A determines its efficacy in complementing the yeast DDT defect.

### Differential Subcellular Locations of BdUev1s

**FIGURE 7:**
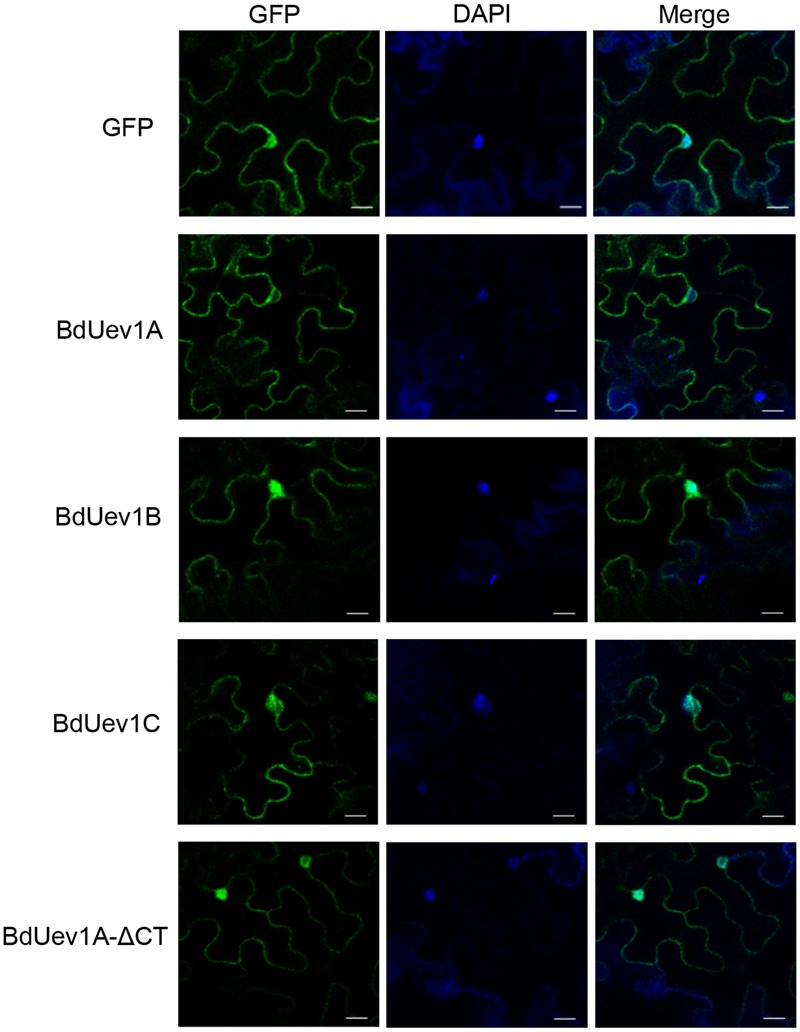
**Subcellular localization of GFP-BdUev1s.** Left panels, transient expression of GFP and GFP-tagged BdUev1s in tobacco epidermal cells visualized by epifluorescence microscopy. Middle panels, DAPI staining to visualize nuclei. Right panels, merge of images from corresponding fluorescence and DAPI staining. Bars = 20 μm.

To further address the difference between *BdUEV1A* and *BdUEV1B/C* in complementing the yeast *mms2* mutation, we entertained a hypothesis that their encoded proteins may have different subcellular distributions in plants. To test this hypothesis, three *GFP-BdUEV1* fusion constructs were made, transiently transfected to tobacco (*N. benthamiana*) leaves by *A. tumefaciens* and their subcellular localization was monitored by fluorescence microscopy. As shown in **Figure 7**, both GFP-BdUev1B and GFP-BdUev1C were primarily found in the nucleus. In sharp contrast, GFP-BdUev1A was excluded from the nucleus, while truncation of its C-terminal 18 amino acids (BdUev1A-ΔCT) results in its redistribution to the nucleus, reminiscent of the subcellular localization patterns of GFP-BdUev1B and GFP-BdUev1C. Since the DDT function must occur in the nucleus, the above observations further suggest that BdUev1B and BdUev1C are involved in plant DNA-damage response while BdUev1A is not. Meanwhile, this observation indicates that the C-terminal tail of BdUev1A plays a critical role in its subcellular localization and physiological functions.

### Expression of *BdUEV1s* during Development and in Response to Abiotic Stresses

**FIGURE 8:**
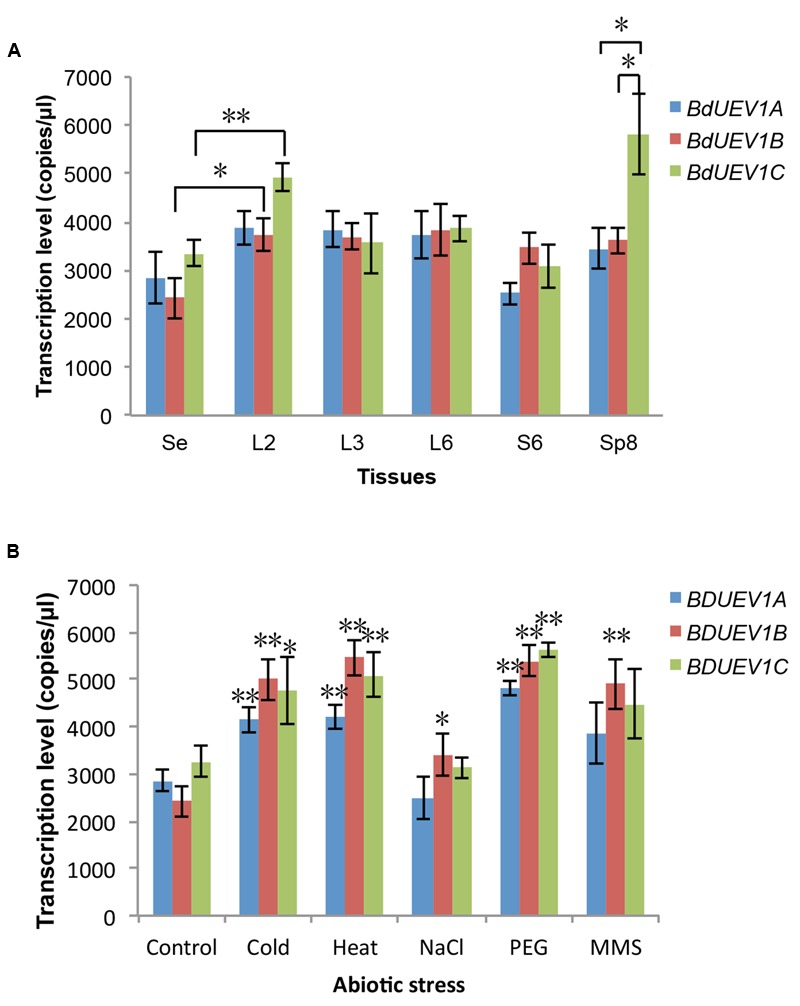
**Quantitative analysis of expression of *BdUEV1*s. (A)** Expression levels of *BdUEV1*s in different tissues during *B. distachyon* development. Samples were taken from different developmental stages and tissues as indicated. Se: 7-day seedling; L2: leaves from 2-week old plants; L3: leaves from 3-week old plants; L6 and S6: leaves and stems from 6-week old plants; Sp8: spikes from 8-week old plants. **(B)** The expression of *BdUEV1*s under different abiotic stresses. Control: 7-day old seedlings; Cold: 7-day old seedlings were treated at 4°C for 12 h; Heat: 7-day old seedlings were treated at 42°C for 12 h; PEG: 7-day old seedlings were treated with 25% PEG for 12 h; MMS: 7-day old seedlings were treated with 0.01% MMS for 12 h. ^∗^*P* < 0.05; ^∗∗^*P* < 0.01.

In a recent study, we found no obvious fluctuation in the expression of *BdUBC13* genes in different tissues or during plant development (Guo et al., 2016). This expression pattern is consistent with *UBC13* genes from *Arabidopsis* (Wen et al., 2006) and rice (Zang et al., 2012). In contrast, the expression of *Arabidopsis UEV1* genes appears to fluctuate in different tissues and during development (Wen et al., 2008). To investigate the *BdUEV1* gene expression during development, total RNA was extracted from 7-day seedlings as well as different tissues and the *BdUEV1* transcript levels were determined by ddPCR. As shown in **Figure 8A**, the three *BdUEV1* genes have a relatively uniform expression in different tissues except for *BdUEV1C* in L2 (leaves from transition phase) and Sp8 (spikes from 8-week old) phases. The *BdUEV1B* expression also has a significant but moderate increase during L2.

We also analyzed *BdUEV1* expression in response to abiotic stress using ddRT-PCR (**Figure 8B**). It is of great interest to note that cold stress induces the expression of all three *BdUEV1*s reminiscent of cold-induced *BdUBC13* expression (Guo et al., 2016). The transcription level of all *BdUEV1*s also significantly increased after heat and PEG treatments. On the other hand, only *BdUEV1B* expression has a twofold increase after MMS treatment, suggesting the involvement of *BdUEV1B* in DNA-damage response.

## Discussion

In this study, we isolated and characterized three *B. distachyon UEV1* genes encoding Ubc-like proteins. Our observations collectively indicate that there are two classes of *UEV* genes in *B. distachyon* as well as other plant species. First of all, genome database analysis shows that all known plant species contain several highly conserved *UEV* genes, in which at least one (e.g., *BdUEV1B* and *BdUEV1C*) encodes a short version of Uev1 and one (e.g., *BdUEV1A*) encodes one with an additional C-terminal tail. Secondly, phylogenetic analysis groups all plant Uevs with the additional C-terminal tail in one branch, indicating that the two *UEV* classes were independently evolved early in higher plants. Thirdly, it appears that *BdUEV1B/C* can fully rescue yeast *mms2* null mutant phenotypes while *BdUEV1A* is only partially functional, and this difference becomes more obvious when *BdUBC13* is used to replace *yUBC13* in yeast cells. Fourthly, we found in this study that BdUev1A and BdUev1B/C have different subcellular distribution patterns in cultured tobacco cells. While BdUev1B/C proteins are primarily located in the nucleus, BdUev1A is excluded from the nucleus, suggesting that their physiological functions are different. Finally, we surprisingly found that when the C-terminal tail is deleted from BdUev1A, it behaves like BdUev1B/C with respect to both functional complementation of the yeast *mms2* mutant and subcellular localization. The above observations along with a previous report on the *AtUEV1D* function (Wen et al., 2008) collectively allow us to speculate that BdUev1B/C are involved in nuclear functions including DNA-damage response, while BdUev1A plays roles in non-nuclear functions. Hence, the reduced ability for *BdUEV1A* to complement the yeast *mms2* mutant is perhaps due to product exclusion from the yeast nucleus. It is interesting to note that plant *UBC13* genes are expressed at a high level and do not fluctuate (Wen et al., 2006; Zang et al., 2012; Guo et al., 2016), and BdUbc13 is distributed to both nucleus and cytoplasm, as well as the cytoplasmic membrane (Guo et al., 2016), implying that *UBC13* may serve as a housekeeping gene and different *UEV1* genes encode regulatory subunits to modulate the heterodimer activity through spatial distribution and differential expression. The above envisioned plant *UEV* functions are reminiscent of the mammalian *UEV*s, in which *UEV1A* encodes a Uev with a unique 25-aa N-terminal sequence (Xiao et al., 1998). While both Uev1A and Mms2 are able to form stable complexes with Ubc13 and promote Lys63-linked polyubiquitination, they have distinct *in vivo* functions: Mms2 is involved in DNA-damage response but not NF-κB signal transduction, whereas Uev1A is required for the NF-κB activation but not for DNA-damage response (Andersen et al., 2005). Furthermore, expression of *UEV1C*, a *UEV1* splicing variant lacking the *UEV1A* sequence coding for its N-terminal unique region, failed to confer NF-κB activation (Wu et al., 2014).

Lys63-linked polyubiquitination is found to be involved in several important cell signaling pathways, including DDT (Brusky et al., 2000), DNA double-strand break repair (Thorslund et al., 2015), inflammation and immunity (Deng et al., 2000; Chang et al., 2012), protein endocytosis (Marchal et al., 2000; Soetens et al., 2001), mitochondrial inheritance (Fisk and Yaffe, 1999) and in ribosome function (Spence et al., 2000). Ubc13 is the only known E2 dedicated to catalyzing Lys63-linked Ub chain assembly and this activity absolutely requires Uev as a cofactor (Hofmann and Pickart, 1999; McKenna et al., 2001). While plant Ubc13 has been shown to be involved in several cellular processes, in the majority of cases, its cognate E3 is unclear and whether it requires Uev as a cofactor is not known. We anticipate that if Lys63-linked polyubiquitination of the target protein is required for the Ubc13 function, it must need a Uev as a cofactor. In this respect, it is of great interest to determine which *UEV1* gene(s) is required for these processes.

## Author Contributions

Experimental design: HG, RW, and WX; Experiments: HG, RW, and QW; manuscript preparation: HG and WX; Supervision, funding, and reagents: WX and RD.

## Conflict of Interest Statement

The authors declare that the research was conducted in the absence of any commercial or financial relationships that could be construed as a potential conflict of interest.
