# Supplementary material for: Three Brachypodium distachyon Uev1s Promote Ubc13-Mediated Lys63-Linked Polyubiquitination and Confer Different Functions
Source: Front Plant Sci. 2016 Oct 18;7:1551. doi: 10.3389/fpls.2016.01551 (PMC5067413; doi:10.3389/fpls.2016.01551)
Supplement: Supplementary file 1 [file Data_Sheet_1.pdf]

SUPPLEMENTARY TABLES AND FIGURES

Table S1. ***Saccharomyces cerevisiae* strains**

Strain	Genotype	Source
PJ69-4A	*MATa trp1-901 leu2-3,112 ura3-52 his3-200 gal4Δ gal80 Δ Met2::GAL7-lacZ LYS2::GAL1-HIS3 GAL2-ADE2*	P. James
HK578-10D	*MATa ade2-1 can1-100 his3-11,15 leu2-3,112 trp1-1 ura3-1*	H. Klein
WXY902	HK578-10D with *mms2Δ::HIS3*	Lab stock
WXY955	HK578-10D with *mms2Δ::HIS3 ubc13Δ::hisG-URA3-hisG*	Lab stock
DBY747	*MATa his3-1 leu2-3, 112 trp1-289 ura3-52*	D. Botstein
WXY642	DBY747 with *mms2Δ::HIS3*	Lab stock

TABLE S2. Primers used for plasmid construction

plasmid	Forward Primers	Reverse Primers
pGAD-UEV1A	CCGGAATTCATGGGGTCCGAGGGATCCG	ACGCGTCGACTTACATAATGACACATCTAATAG
pGBT9-UEV1A		
pGEX6p-1-UEV1A		ACGCGTCGACTTAACCTTCATGAGGCTGGTGG
pGAD-UEV1A-ΔCT		
pGAD-UEV1B	CCGGAATTCATGGCGTCGAGCGGCGACGC	ACGCGTCGACTTAGCCGTAGAATGTCCCTTCTG
pGBT9-UEV1B		
pGEX6p-1-UEV1B		
pGAD-UEV1C	CCGGAATTCATGACGCTGGGCAGCTCCG	ACGCGTCGACCTAGAAGAACGTCCCTTCAGG
pGBT9-UEV1C		
pGEX6p-1-UEV1C		
pCAMBIA1300-GFP-UEV1A	ACCGAGCTCATGGGGTCCGAGGGATCCGC	CGGGGTACCTTACATAATGACACATCTAATAGC
pCAMBIA1300-GFP-UEV1A-ΔCT		ACGCGTCGACTTAACCTTCATGAGGCTGGTGG
pCAMBIA1300-GFP-UEV1B	ACCGAGCTCATGGCGTCGAGCGGCGACGC	CGGGGTACCTTAGCCGTAGAATGTCCCTTC
pCAMBIA1300-GFP-UEV1C	ACCGAGCTCATGACGCTGGGCAGCTCCGG	CGGGGTACCCTAGAAGAACGTCCCTTC

TABLE S3. Primers used for ddPCR

gene	Forward Primers	Reverse Primers
BdUEV1A	5'-CTCGAATTCAAATGGGG TCC GAGGGATCC	5'-CAGTGAGCTCTTACATAATGACACATCTAATAG
BdUEV1B	5'-CTCGAATTCAAATGGCG TCG AGCGGCGAC	5'-CAGTGAGCTCTTAGCCGTAGAATGTCCCTTC
BdUEV1C	5'-CTCGAATTCAAATGACGCTGGGCAGCTCCG	5'-CAGTGAGCTCCTAGAAGAACGTCCCTTCAGG

FIGURE S1. His_6-rhUB(K63R) has background bands. The ubiquitination reactions were performed as described in Figure 3 except that the absent components were replaced by the corresponding protein solution buffer. After the reactions, samples were subjected to SDS-PAGE and Western blotting using an anti-Ub antibody to monitor whether His_6-rhUB(K63R) has background bands. rhUb, recombinant human ubiquitin. Asterisks indicate the His_6-rhUB(K63R) background bands in lanes 1, 2 and 3.

FIGURE S2. Functional complementation of the yeast *ubc13 mms2* null mutant by *BdUEV1*s and *BdUBC13B*. (**A**) Complementation of the *ubc13/mms2* double mutant by *BdUEV1*s and *BdUBC13B*. Yeast strain HK578-10D (wild type) and the *ubc13*Δ *mms2*Δ transformants were grown overnight and then printed onto the gradient plate. The YPD control (left) and YPD+0.025% MMS gradient (right) plates were incubated at 30°C for 3 days. Arrow points to gradually increasing MMS concentration. (**B**) Functional complementation of the *ubc13/mms2* double mutant by *BdUEV1s* and *BdUBC13B* using representative DNA-damaging agents by a serial dilution assay. Yeast strains as indicated were grown overnight in SD selective media, diluted and treated with the DNA-damaging agents.

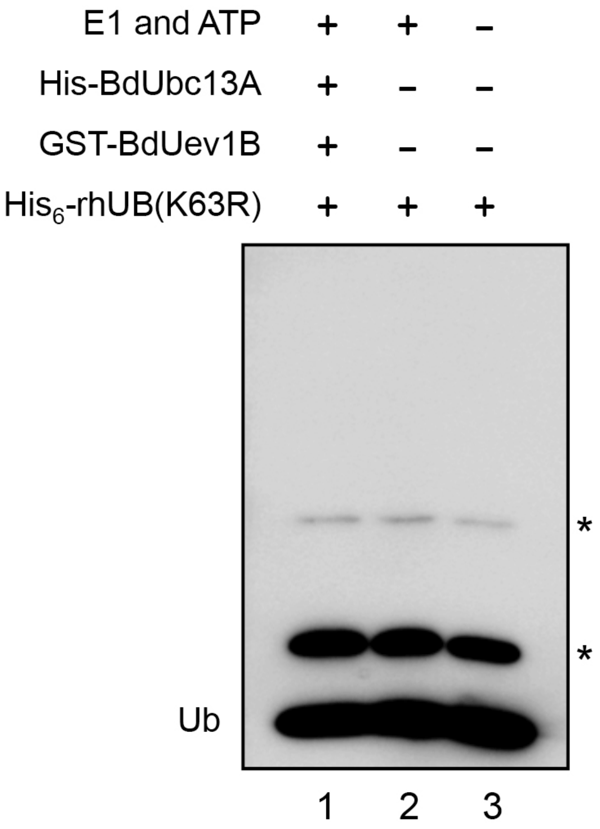

Figure S2

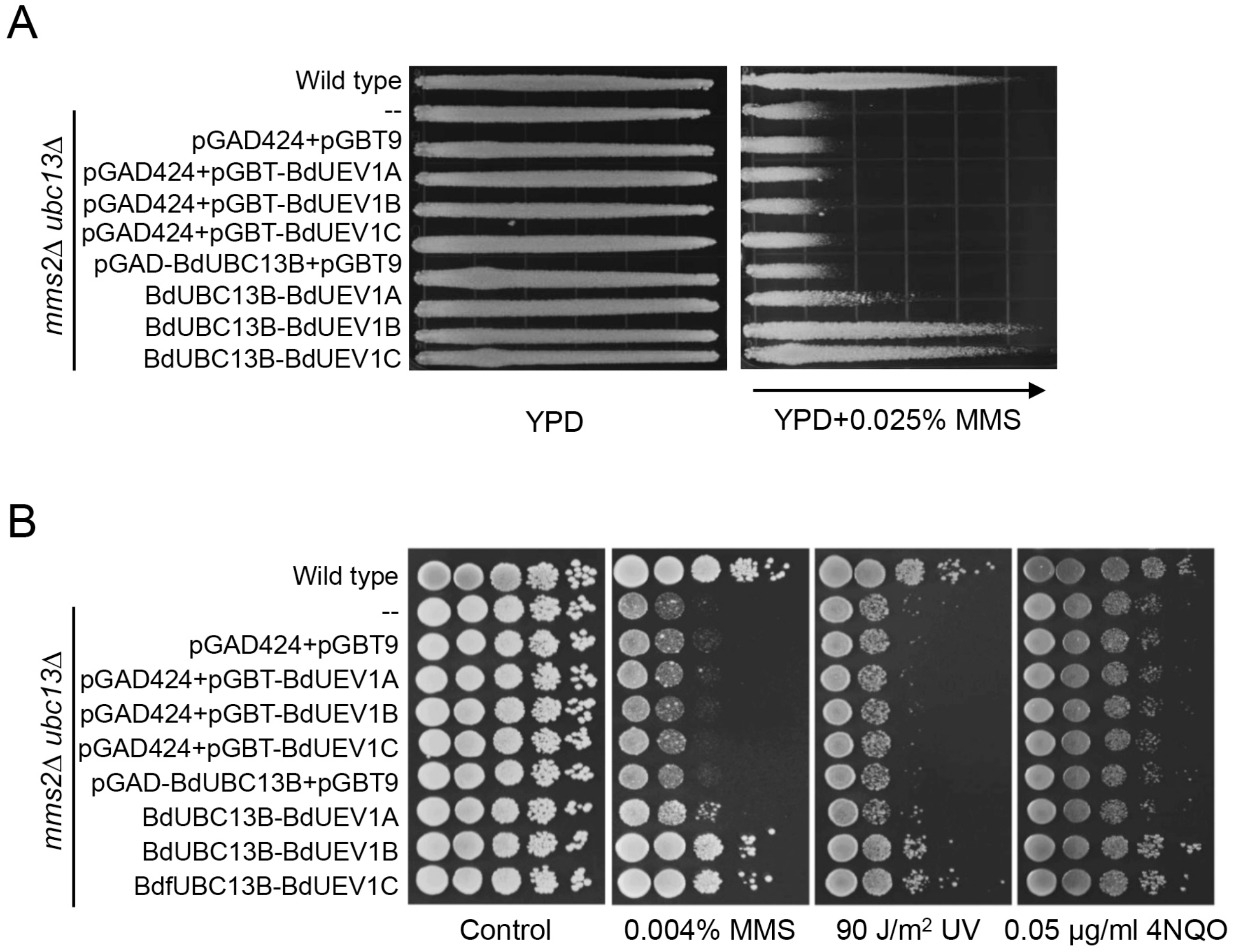
A
Wild type
--
pGAD424+pGBT9
pGAD424+pGBT-BdUEV1A
pGAD424+pGBT-BdUEV1B
pGAD424+pGBT-BdUEV1C
pGAD-BdUBC13B+pGBT9
BdUBC13B-BdUEV1A
BdUBC13B-BdUEV1B
BdUBC13B-BdUEV1C
mms2Δ ubc13Δ
YPD
YPD+0.025% MMS
B
Wild type
--
pGAD424+pGBT9
pGAD424+pGBT-BdUEV1A
pGAD424+pGBT-BdUEV1B
pGAD424+pGBT-BdUEV1C
pGAD-BdUBC13B+pGBT9
BdUBC13B-BdUEV1A
BdUBC13B-BdUEV1B
BdfUBC13B-BdUEV1C
mms2Δ ubc13Δ
Control
0.004% MMS
90 J/m2 UV
0.05 µg/ml 4NQO

Figure S2
